# Analysis of *Schistosoma mansoni* Extracellular Vesicles Surface Glycans Reveals Potential Immune Evasion Mechanism and New Insights on Their Origins of Biogenesis

**DOI:** 10.3390/pathogens10111401

**Published:** 2021-10-29

**Authors:** Maude Dagenais, Jared Q. Gerlach, George R. Wendt, James J. Collins, Louise E. Atkinson, Angela Mousley, Timothy G. Geary, Thavy Long

**Affiliations:** 1 Institute of Parasitology, McGill University, Ste-Anne-de-Bellevue, QC H9X 3V9, Canada; timothy.g.geary@mcgill.ca (T.G.G.); thavy.long@mcgill.ca (T.L.); 2 Glycoscience Group, Advanced Glycoscience Research Cluster, National Centre for Biomedical Engineering Science, National University of Ireland Galway, H91 TK33 Galway, Ireland; jared.gerlach@nuigalway.ie; 3 Department of Pharmacology, University of Texas Southwestern Medical Center, Dallas, TX 75390, USA; george.wendt@utsouthwestern.edu (G.R.W.); jamesj.collins@utsouthwestern.edu (J.J.C.III); 4 Microbes and Pathogen Biology, The Institute for Global Food Security, School of Biological Sciences, Queen’s University-Belfast, Belfast BT9 5DL, UK; l.atkinson@qub.ac.uk (L.E.A.); a.mousley@qub.ac.uk (A.M.)

**Keywords:** extracellular vesicles, exosomes, secretome, helminths, glycans, schistosomes, sialic acid, lectin histochemistry, lectin microarray

## Abstract

Parasitic helminths are master manipulators of host immunity. Their strategy is complex and involves the release of excreted/secreted products, including extracellular vesicles (EVs). The protein and miRNA contents of EVs have been characterised for many parasitic helminths but, despite reports suggesting the importance of EV surface carbohydrate structures (glycans) in the interactions with target cells and thus subsequent effector functions, little is known about parasite EV glycomics. Using lectin microarrays, we identified several lectins that exhibit strong adhesion to *Schistosoma mansoni* EVs, suggesting the presence of multiple glycan structures on these vesicles. Interestingly, SNA-I, a lectin that recognises structures with terminal sialic acid, displayed strong affinity for *S. mansoni* EVs, which was completely abolished by neuraminidase treatment, suggesting sialylation in the EV sample. This finding is of interest, as sialic acids play important roles in the context of infection by aiding immune evasion, affecting target recognition, cell entry, etc., but are not thought to be synthesised by helminths. These data were validated by quantitative analysis of free sialic acid released from EVs following treatment with neuraminidase. Lectin histochemistry and fluorescence in situ hybridisation analyses on whole adult worms suggest the involvement of sub-tegumental cell bodies, as well as the digestive and excretory systems, in the release of EVs. These results support previous reports of EV biogenesis diversity in trematodes and potentially highlight new means of immune modulation and evasion employed by schistosomes.

## 1. Introduction

Schistosomiasis is caused by infection with parasitic trematodes in the genus *Schistosoma*, which have a complex life cycle that includes freshwater snails as intermediate hosts and mammalian definitive hosts. Despite mass drug administration programs with praziquantel, schistosomiasis is estimated to affect ~240 million people worldwide [1] and to still be responsible for the loss of 1.43 million disability-adjusted life years [2].

Owing to their ability to evade and modulate host immune responses [3,4], adult schistosomes can survive within their mammalian host for many years [5]. In this regard, their excreted/secreted products (ESPs) are of particular interest, as parasite-secreted molecules are instrumental for host-parasite interactions and thus represent promising targets for diagnostic and vaccine antigen discovery [4,6,7,8]. Significant advances have been made in the characterisation of the secretomes of *Schistosoma* species [9,10,11], but it is only recently that the release of extracellular vesicles (EVs) by schistosomes has been described [8,12,13,14,15,16], suggesting such vesicles as a route of ESP secretion.

EVs include all types of secreted membrane-bound vesicles, of which exosomes (formed within multivesicular bodies) and microvesicles (formed by the outward budding of the plasma membrane) are the best characterised [17]. Despite mounting evidence for vesicle-based secretion in schistosomes, the specifics of EV release remain elusive and the origin(s) of EV secretion is still unclear. There is increasing evidence for the release of EVs in parasitic infections, with roles in both parasite-parasite inter-communication [18] and parasite-host interactions [7,19,20]. EVs transfer molecules from one cell to another via membrane vesicle trafficking, thus explaining the broad array of functional activities attributable to them. However, little is known about the mechanisms of parasite-derived EV uptake by host cells and therefore how their effects are transduced in vivo. This is due, at least in part, to the lack of knowledge regarding schistosome-derived EV surface molecules. A growing body of evidence suggests a requirement for various carbohydrate moieties, on the surface of both cells and vesicles, for EV uptake by recipient cells [21,22,23,24,25]. Despite increasing appreciation for the role of glycans in EV biology, little information is available regarding carbohydrate structures present on the surface of adult *S. mansoni*-derived EVs. Glycoproteins secreted by helminth parasites are immunogenic and represent appealing components of vaccine preparations. A better understanding of the glycans present on vesicles secreted by the parasite could lead to the identification of novel biomarkers for the development of superior diagnostic tools as well as new targets for prevention and therapy of schistosomiasis.

We previously characterised the protein and miRNA composition of adult *S. mansoni*-derived EVs [12]. Here, we profiled the major glycan motifs present on the surface of adult *S. mansoni-*derived EVs using lectin microarrays. Interestingly, SNA-I, a lectin that recognises structures with terminal sialic acid residues, displayed strong affinity for *S. mansoni* EVs, which was validated by quantitative analysis of free sialic acid released following treatment with neuraminidase, suggesting sialylation in the EV sample. The presence of sialic acid in *S. mansoni* EV samples is of interest, as sialic acids play important roles in infection by, for example, aiding immune evasion, and affecting target recognition and cell entry [26,27], but are not thought to be synthesised by helminths [28]. Moreover, our lectin histochemistry and fluorescence in situ hybridisation (FISH) studies suggest possible roles for the tegument, the gut and the excretory system in the secretion of EVs, indicating the presence of a heterogeneous EV population.

## 2. Results

### 2.1. Sialic Acid Residues Are Detected on Schistosoma mansoni-Derived EVs

**Figure 1:**
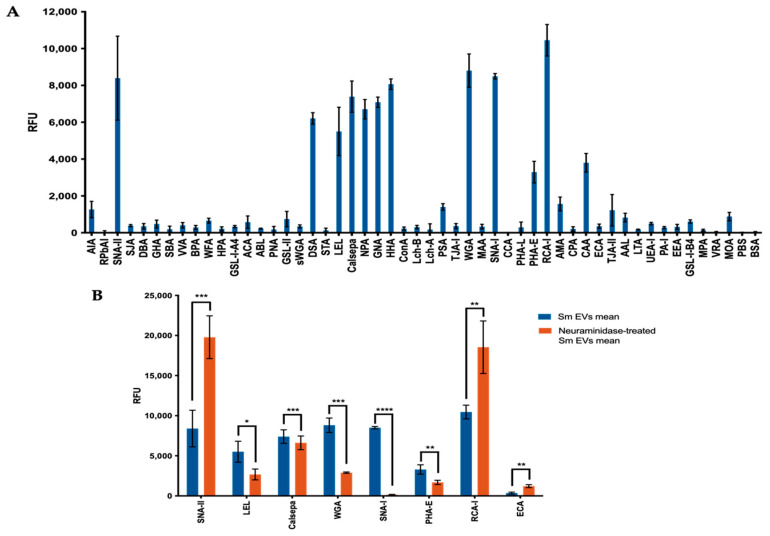
Lectin microarray. Microarray slides were incubated with PKH26-labelled EVs and imaged at 532 nm in an Agilent G2505B microarray scanner at 100% PMT (**A**) Lectin microarray response generated for *S. mansoni* EVs. (**B**) Significant effect of neuraminidase treatment on lectin-binding at the surface of EVs. Data subjected to total intensity mean normalisation, n = 4, +/−SD. Statistics were performed using a paired *t*-test * *p* > 0.05, ** *p* > 0.01, *** *p* > 0.0005, **** *p* > 0.00001.

Using lectin microarrays, we identified several lectins with various binding specificities (Table 1; see Supplemental Table S1 for full list of lectins and specificities) that exhibited strong adhesion with EVs, including SNA-II, DSA, LEL, Calsepa, NPA, GNA, HHA, WGA, SNA-I, RCA-I and CAA (Figure 1A).

These results suggest the presence of a wide range of glycan structures on these vesicles. Interestingly, SNA-I, a lectin that recognises structures with α2-6-linked terminal sialic acid, displayed strong affinity for *S. mansoni* EVs, which treatment with a broad-spectrum neuraminidase (also “sialidase”; enzymes that cleave sialic acid) completely abolished (Figure 1B; Table 2), suggesting the presence of oligosaccharides with terminal sialic acid residues in the EV sample. WGA, a lectin that recognises both N-acetyl-glucosamine (GlcNAc) and sialic acid residues on proteins, also demonstrated a sharp decrease in intensity after EVs were treated with neuraminidase. Importantly, SNA-II and RCA-I, which have affinity for structures which are frequently terminated with sialic acids (those with distal *N*-acetyl-galactosamine and/or galactose, especially in Type I and Type II N-acetyllactosamine configurations) demonstrated sharp increases in signals following neuraminidase treatment (Figure 1B; Table 2), which is consistent with the exposure of these structures following removal of terminal sialic acids.

**Figure 2:**
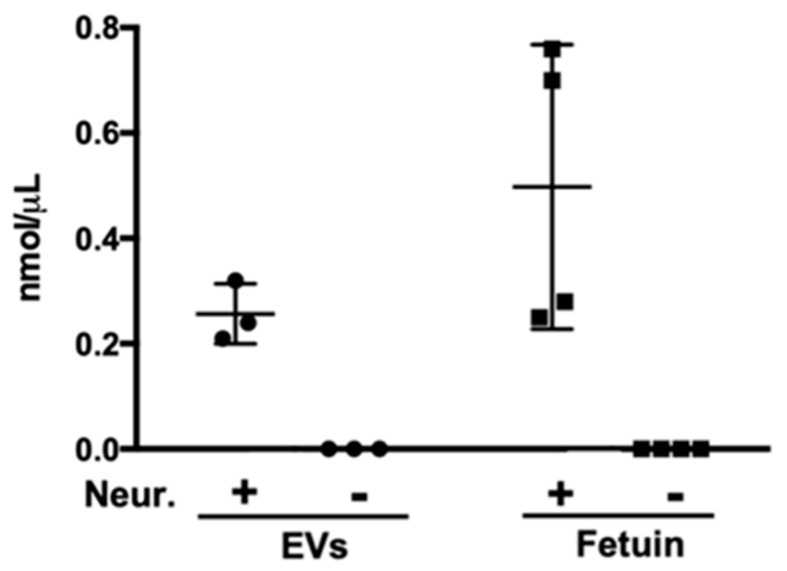
Treatment of *S. mansoni* EVs with neuraminidase (Neur.) results in the release of free sialic acid residues. EVs were incubated with a broad-spectrum neuraminidase, after which samples were assayed for the presence of free sialic acid residues. Fetuin was used as a control sialylated glycoprotein. +/−SD.

We reasoned that if terminal sialic acid residues were indeed present on the surface of EVs, not only would hydrolysis of these structures by a broad-spectrum neuraminidase prevent SNA-I binding, but would also result in the release of free sialic acid. We thus validated sialylation of EVs by quantitative analysis of sialic acid released following treatment with neuraminidase and observed a consistent increase in free sialic acid concentration as a result of enzymatic digestion (Figure 2).

**Figure 3:**
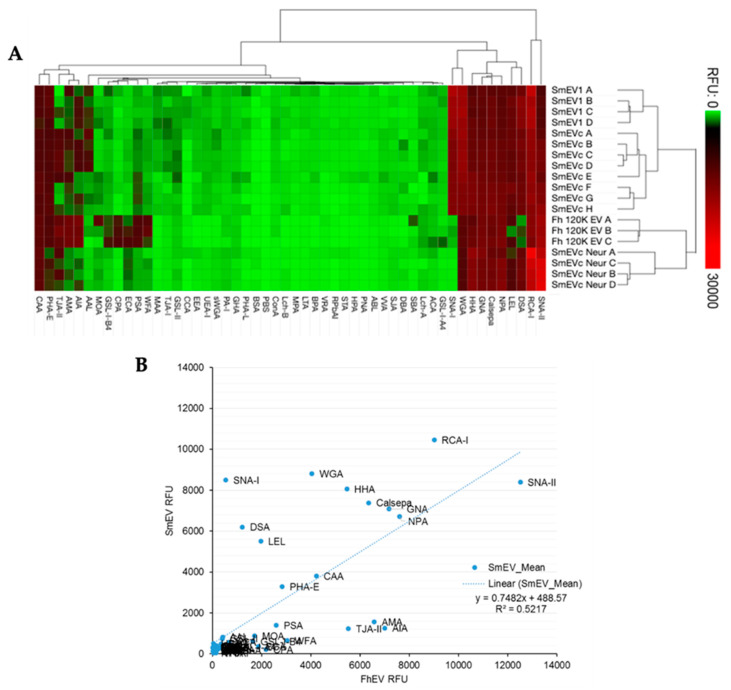
Comparison of *Schistosoma mansoni*-derived EVs and *Fasciola hepatica*-secreted EVs lectin microarray profiles. (**A**) Heat map and two-dimensional hierarchical clustering of scale-normalised lectin microarray profile data for all twelve *Sm*EV technical replicates, 4 from one EV isolation sample (SmEV1 A-D) and 8 from pooled EV samples (*Sm*EVc A-H), and three *Fh*EV replicates (*Fh* 120k EV A-C) obtained by de la Torre-Escudero et al. (2019). Data depicted in heat map was scaled to fit a 0–30,000 RFU window and clustered by average linkage, Euclidean distance method (**B**) Regression analysis comparing *S. mansoni* EV and *F. hepatica* EV lectin-binding profiles.

Microarray profiles were nearly identical between technical replicates from two independent samples (*Sm*EV1 and *Sm*EVc; Figure 3A). We compared our analysis to the EV microarray profile of another trematode, *Fasciola hepatica*, obtained by de la Torre-Escudero et al. [21]. A two-dimensional hierarchical clustering of scale-normalised lectin microarray profile data for all technical replicates of the two trematode genera highlighted the different abundances and/or combinations of carbohydrate moieties present on the surface of their respective EVs (Figure 3A). The four mannose-binding lectins which displayed strong affinity with *S. mansoni* EVs, Calsepa, NPA, GNA and HHA maintained a comparably strong signal with *F. hepatica* EVs, suggesting a similar abundance of high-mannose glycans for both trematodes (Figure 3A,B). RCA-I and CAA, with specificity for terminal galactose, also had similar affinity for both EV populations (Figure 1A,B). Overall, *F. hepatica* EVs had a slightly wider variety of lectin affinities than *S. mansoni* EVs, but had very low-level interactions with SNA-I (Figure 3), suggesting minimal sialylation in the *F. hepatica* samples, if any.

### 2.2. Lectin Immunocytochemistry and In Situ Hybridisation Reveal Sub-Tegumental Cells as a Potential Source of EVs in Schistosoma mansoni

**Figure 4:**
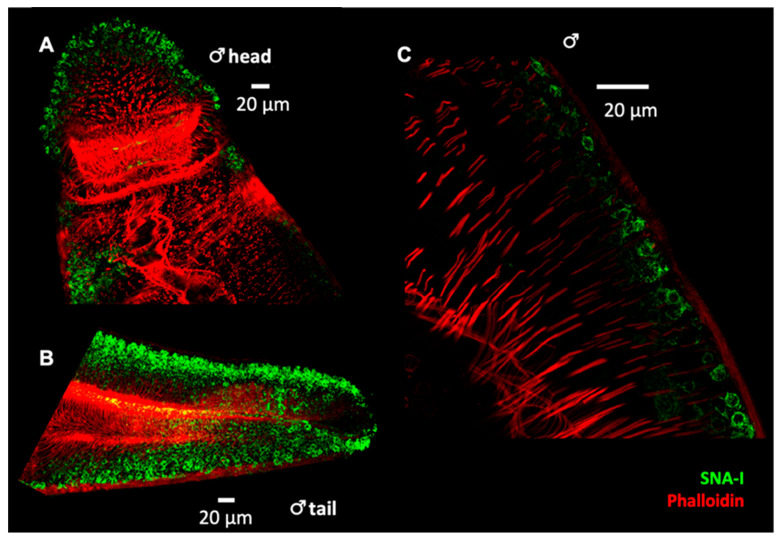
Whole worm labelling with SNA-I lectin. Flat-fixed adult worms were labelled with SNA-I (green) and fluorescent phalloidin (red) and imaged by confocal microscopy (**A**) Transverse plane through the anterior portion of a male worm and (**B**) maximum intensity projection of z-stacks acquired at the tail of a male worm. (**C**) Higher magnification of a transverse plane through the side of the worm.

**Figure 5:**
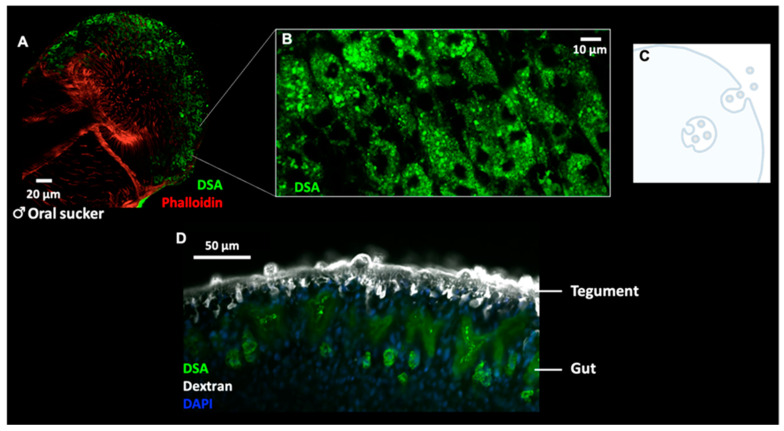
Whole worm labelling with DSA. (**A**,**B**) Flat-fixed adult worms were labelled with DSA (green) and fluorescent phalloidin (red) and imaged by confocal microscopy (**A**) Transverse plane through the oral sucker of a male worm and (**B**) Higher magnification of cells labelled with DSA show a granular labelling pattern. (**C**) Cartoon depicting the biogenesis of exosomes, via the formation of intraluminal vesicles within multivesicular endosomes (MVEs) and the subsequent fusion of MVEs with the plasma membrane, thereby releasing exosomes into the extracellular space. (**D**) Cross-section of a male worm labelled with fluorescent dextran (tegument), DAPI (nuclei) and DSA (green) shows clear labelling of the parasite gut with the lectin.

**Figure 6:**
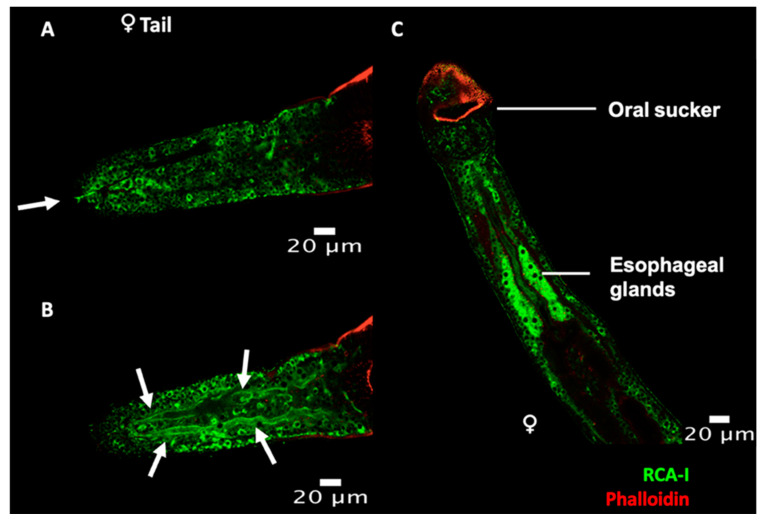
Whole worm labelling with RCA-I. (**A**–**C**) Flat-fixed adult worms were labelled with RCA-I (green) and fluorescent phalloidin (red). (**A**,**B**) Transverse plane acquired at different levels through the tail of a female worm, arrows indicate (**A**) the excretory pore and (**B**) the excretory tubules. (**C**) Image of the anterior region of a female worm.

**Figure 7:**
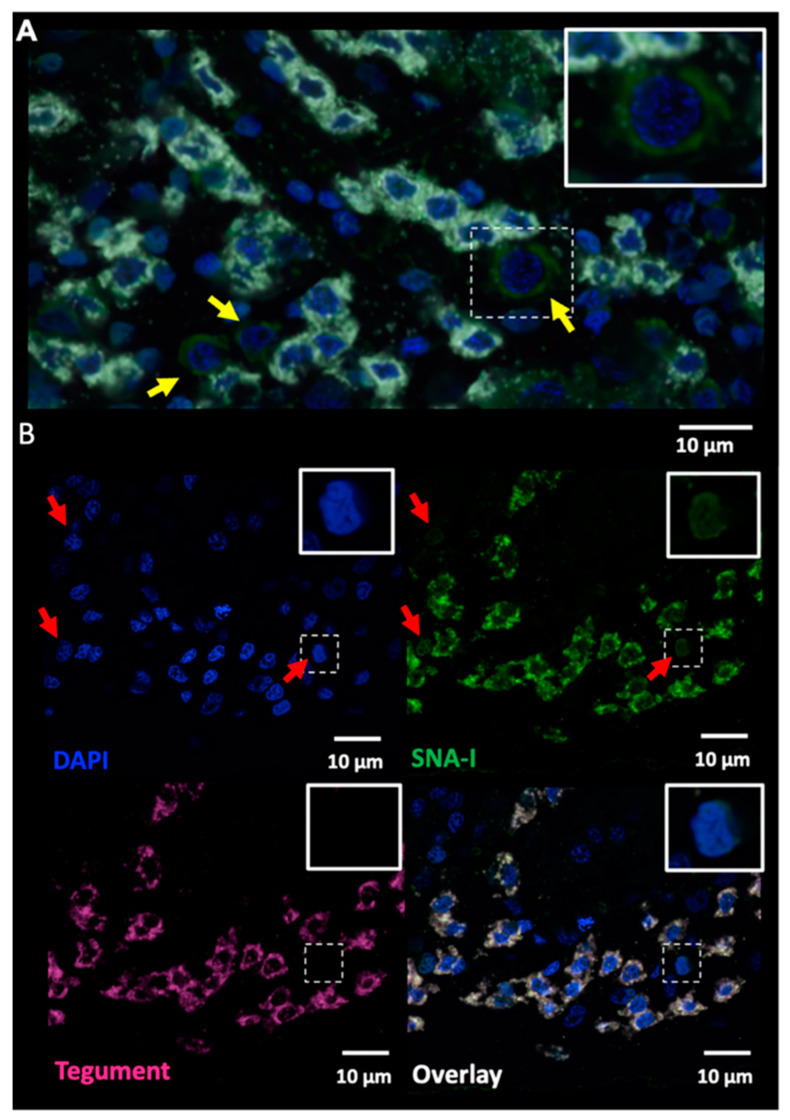
All definitive tegumental cells are SNA-I positive. Fluorescence in situ hybridisation with tegument markers (*calpain*, *gtp-4*, *annexin*, and *npp-5*; white), and SNA-I (green) co-labelling. 100% of tegumental cells are also positive for SNA-I labelling, SNA-I also labelled (**A**) a few non-tegumental cells at the same level within the worm (yellow arrows; inset shows enlargement of an SNA-I^+^ non-tegumental cell) and (**B**) some nuclei (red arrows; insets).

**Figure 8:**
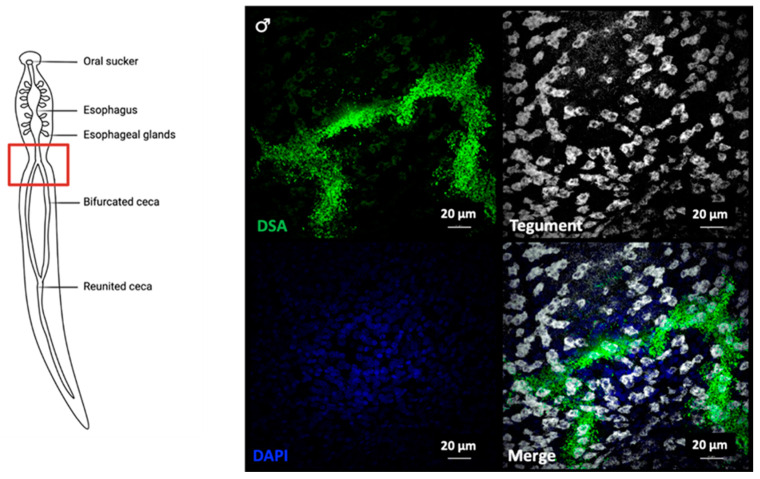
DSA labels definitive tegumental cells and the gut of the parasite. Adult worms were labelled by fluorescence in situ hybridisation with tegument markers (*calpain*, *gtp-4*, *annexin*, and *npp-5*), after which they were incubated with fluorescent DSA lectin and imaged via confocal microscopy. Dorsal view of a male worm, showing the bifurcated intestinal ceca, right above the ventral sucker.

To identify worm structure(s) as possible sources of EVs, we performed histochemistry on whole adult worms with three lectins that exhibited strong affinity for *S. mansoni* EVs (DSA, RCA-I and SNA-I) (Figure 4, Figure 5 and Figure 6). We observed strong labelling of distinct cell bodies immediately below the body wall muscle by SNA-I (Figure 4) and less intense labelling of similar cellular structures by DSA (Figure 5A). We identified these cells as tegumental cell bodies by performing FISH-lectin co-labelling experiments (Figure 7 and Figure 8), using a combination of markers previously shown to specifically label definitive tegumental cells (*calp*, *gtp-4*, *npp-5*, *annexin*) [29]. Indeed, 100% of FISH-labelled tegumental cells were positive for SNA-I (Figure 7) and DSA labelling (Figure 8). A few non-tegumental cells at the same level within the worm were also SNA-I positive (possibly tegumental progenitor cells; Figure 7A). Interestingly, we also noted SNA-I nuclear labeling, not only in tegumental cell bodies, but also in nuclei at the same level within the worm (Figure 7B).

Higher magnifications of DSA-labelled tegumental cells revealed a granular pattern of DSA localisation (Figure 5B), which could indicate the presence of intraluminal vesicle (ILV)-filled multivesicular bodies (MVBs; Figure 5C). In addition, DSA strongly labelled the gut of the parasite (Figure 5D and Figure 8), marking the digestive system as a potential route of EV secretion in adult worms.

Finally, RCA-I labelled various structures within the worm, including potential tegumental cells and what appear to be the excretory pore and excretory tubules (Figure 6A,B), which might implicate the excretory system in the release of at least a subset of EVs. RCA-I also labels the esophageal glands (Figure 6C).

## 3. Discussion

There is growing interest for understanding the role of EVs in the context of parasitic infections. Many groups have studied the protein, miRNA, and even lipid composition of different populations of parasite EVs, providing important information regarding pathways of EV biosynthesis and potential functions [8,12,16,30,31,32,33]. Despite mounting evidence pointing to the importance of the glycan composition of EVs [21,22,23,34], only one study on schistosome EV glycomics has been published [25]. Glycan structures coating the surface of EVs are likely to be functionally important to host-parasite interactions, as they are presumably responsible for delivering parasite-derived molecules to target cells. In this study, we profiled various carbohydrate moieties present on the surface of adult *S. mansoni* EVs via lectin binding. Comparison with lectin profiling of *F. hepatica* EVs [21] revealed a comparable abundance of high-mannose sugars, as well as the presence of terminal galactose on EVs from both trematodes.

Our results also suggest the presence of sialic acid residues on adult *S. mansoni*-derived EVs. Helminths are generally regarded as unable to synthesise sialic acids due to their apparent lack of enzymes required for sialic acid synthesis [28,35]. However, evidence of sialylation has been found in different helminth glycoprotein preparations, such as the *Echinococcus granulosus* hydatid cysts [36], *Taenia crassiceps* metacestodes [37], and *F. hepatica* tegument [38], but these findings have systematically been attributed to the presence of host glycans. The origin of sialic acid in our samples remains unclear (parasite, host, or EV-depleted fetal bovine serum; FBS), but the presence of host or bovine sialylated molecules on *S. mansoni* EVs, despite the many washes and a density sedimentation step, could be relevant, given that in vivo, EVs are released in host blood and would be exposed to similar blood proteins. We previously found no evidence of murine or bovine miRNAs in our EV samples [12], suggesting that minimal contamination of schistosome EVs with host or bovine EVs occurred during our EV isolation protocol.

Coating with host glycoproteins could confer EVs protection from host immune effectors, allowing them to travel undetected and interact with target cells to exert their function. Indeed, previous lectin microarray analyses on mammalian EVs revealed an enrichment in specific glycan features, including α2-6 linked sialic acid, compared to the plasma membrane of the producing cells [23]. Additional reports have also linked sialic acid residues and sialic acid receptors to EV uptake [22,24,39] and others have observed the impact of EV desialylation on biodistribution in vivo [34], further highlighting the importance of EV glycosylation state. *Fasciola hepatica* EVs, on the other hand, displayed low-level interactions with SNA-I [21], suggesting minimal sialylation. This discrepancy, however, could be explained by the life cycle differences between these parasites; adult *S. mansoni* are found in the blood whilst *F. hepatica* reside in the bile duct. Alternatively, the discrepancy may be due to the fact that liver fluke EVs were collected from parasites cultured without serum [21].

Moreover, given their avidity for *S. mansoni* EVs, we employed SNA-I, DSA and RCA-I in lectin immunofluorescence studies to locate site(s) of EV secretion in adult parasites. SNA-I and DSA both label sub-tegumental cell bodies in adult *S. mansoni*. In addition, we noted SNA-I labelling in a small number of non-definitive tegumental cells as well as some SNA-I positive nuclei at the same level within the worm. Significantly, we observed distinct granular structures in DSA-labelled tegumental cells, perhaps representing future exosomes encapsulated within MVBs, although it is important to note that the size of EVs is below the level of resolution of the confocal instruments used. Besides accumulating within tegument cells, DSA also strongly labelled the gut of the parasite. Lastly, RCA-I labelled various regions of the worm, possibly including tegumental cells, although co-localisation experiments have not been conducted in this case. RCA-I also accumulated in what appears to be the excretory pore and the excretory tubules and around the esophageal glands. Together, these results may indicate the presence of multiple EV sub-populations and the involvement of the tegument and the digestive and protonephridial (excretory) systems in the release of EVs from adult schistosomes. A previous study on adult *F. hepatica* identified the excretory system as the source of small EVs (EVs sedimenting at 120k× *g*; 120k EVs) and gastrodermal epithelial cells lining the gut as the source of large EVs (EVs sedimenting at 15k× *g*; 15k EVs) [21]. The same group also reported localisation of RAL-A (one of two markers used for 120k EVs) in the tegument syncytium and sub-tegumental cells [21]. It is important to consider that the localisation studies performed by de la Torre-Escuerdo et al. were done using antisera raised against markers for specific EV sub-populations (120k and 15k EVs) as their protein content differed, but that lectin microarray profiles of the 120k and 15k EVs were nearly identical. Comparable EV sub-populations (120k and 15k) have also been identified in adult *S. mansoni* [32]; it is thus fair to expect that lectin profiling could be similar between our EV samples and larger 15k EVs. That being said, it is possible that different EV sub-populations have different origins within the parasite, as observed in *F. hepatica*, and that our EV population of interest may only be released by one of the identified structures in our lectin histochemistry experiments.

To better understand the role of EVs in the course of infection, we must start with unravelling their mechanism of action, including how they travel within the host to reach their target cells and how they interact with these cells. EV surface-coating molecules such as glycans are highly likely to be key players in these important steps. Thus, their characterisation is crucial and could lead to the development of novel therapeutic interventions. In addition, glycans present on parasite-derived EVs may be used as biomarkers for the development of superior diagnostic tools.

## 4. Materials and Methods

### 4.1. Parasites

*Biomphalaria glabrata* snails infected with the *S. mansoni* Puerto Rican strain PR1 were maintained the Institute of Parasitology of McGill University. *Schistosoma mansoni* adult worms were obtained by perfusion of female CD1 mice 7 weeks after infection via tail exposure to approximately 150 cercariae. Mice were housed in the animal facility at the Small Animal Research Unit of McGill University as per the McGill University Animal Care Committee (Permit # 2019-8138). Briefly, mice were euthanized by CO_2_ asphyxiation followed by cervical dislocation and adult *S. mansoni* worms harvested by cardiac perfusion. For histochemistry, worms were washed in sterile PBS and fixed immediately (as described below), whereas for EV isolation, worms were maintained in RPMI-1640 medium supplemented with 100 U penicillin, 100 mg/mL streptomycin, and 10% EV-depleted FBS. EV depletion was performed by ultracentrifugation of FBS under sterile conditions for 18 h at 120,000× *g* and 4 °C, followed by filtration through 0.22 μm hydrophilic PVDF Durapore membranes (EMD Millipore; SVGV01015) as described (Kornilov, et al. [40]. FBS was of the highest grade and lowest endotoxin level (Life Technologies; Ref: 16,000). Adult male and female worm pairs were maintained in 6-well plates for 72 h at a density of approximately 20 worms (10 males and 10 females)/well in 10 mL culture medium at 37 °C. Conditioned medium was collected after 48 and 72 h. Worms remained active during this period of incubation, with no apparent change in morphology or movement.

### 4.2. Isolation of Adult S. mansoni Extracellular Vesicles

EVs were isolated as described [12]. Briefly, parasite-conditioned culture medium was collected and centrifuged at increasing speeds: 300× *g*/15 min, 700× *g*/15 min and 3000× *g*/15 min to remove larger debris, and the supernatant was centrifuged at 12,000× *g* for 45 min. Supernatants were filter-sterilised using a 0.22 µm hydrophilic PVDF Durapore membrane as above and centrifuged at 100,000× *g* for 2 h using a Beckman-Coulter SW-48 rotor on a Beckman-Coulter ultracentrifuge. The pellet was re-suspended in sterile GIBCO^®^ Dulbecco’s Phosphate-Buffered Saline (DPBS) (Life Technologies) and loaded onto a sucrose step gradient (25%, 30% and 35%), then centrifuged at 120,000× *g* for 18 h using a SW-48 rotor. Following centrifugation, the 30% sucrose fraction was collected and washed by diluting 4–5-fold with sterile DPBS and EVs were re-pelleted by centrifugation in an SW-28 rotor at 100,000× *g* for 2 h. The pellets were washed once again by re-suspension in DPBS and centrifugation in a SW-41 rotor at 100,000× *g* for 2 h. Finally, pellets were re-suspended in 1.0 mL DPBS, transferred to 1.5 mL Beckman-Coulter ultracentrifuge tubes and centrifuged in an Optima TL100 tabletop ultracentrifuge (Beckman-Coulter) for 1.5 h at 100,000× *g* using a TLA-100.3 rotor. Exosomes were quantified based on protein concentration using a Pierce BCA Protein Assay Kit (ThermoFischer Scientific) and absorbance measured at 562 nm using a Synergy H4 Hybrid plate reader. The pelleted material was snap-frozen and stored at –80 °C.

### 4.3. Soluble Worm Antigen Preparation

Schistosome soluble worm antigen preparation (SWAP) was prepared from ~30 adult worms collected as described above. Worms were freeze-thawed in 150 μL lysis buffer (1X DPBS, 0.5% Triton X-100) with cOmplete^™^ Mini Protease Inhibitor Cocktail (Roche, Laval, QC, Canada), homogenised using a pestle, and centrifuged at 4000× *g* for 30 min at 4 °C. The supernatant was collected and used as SWAP. SWAP concentration was determined using a Pierce BCA Protein Assay Kit as above.

### 4.4. Profiling of Fluorescently-Labeled EVs with Lectin Microarrays

Microarrays (v2.4.0) comprised of 50 unique plant and fungal lectins were printed by a non-contact method onto Schott Nexterion^®^ H (Schott, Mainz, Germany, Cat. No. 1070936) substrates with a Scienion SciFlexArrayer S3 piezoelectric spotter (Scienion, Berlin, Germany) as previously described [41]. Lectins and their specificities are presented in Supplementary Table S1. Microarrays were washed with PBS containing 0.05% Tween 20 (PBST) three times and once with PBS, centrifuged dry (450× *g*, 5 min) and stored at 4 °C with desiccant until use.

Aliquots of EVs were thawed over ice and immediately labeled with the lipophilic dye PKH26 (Sigma Aldrich, Dublin, Ireland) as previously reported [42]. All steps were carried out at room temperature and in the dark. Following labeling, excess dye was removed from EVs by centrifugal filtration in a 500 μL, 100 kDa MWCO spin device (Amicon, EMD-Millipore, Cork, Ireland). The centrifugal filter was pre-washed by centrifugation with PBS containing 0.1% BSA before the entire final volume of the EV labeling mixture was added. An additional 400 µL PBS was added prior to centrifugation for 15 min, 8000× *g*, at room temperature. Finally, an additional 400 µL PBS was added to the spin device prior to the final centrifugation at 8000× *g* in which the final volume was reduced to approximately 50 μL. Concentrated EVs were removed by pipetting and the bottom of the spin device was rinsed with an additional 20 μL PBS which was added to the recovered volume. EVs were used immediately after labeling.

Lectin microarray interrogation of EV surface glycosylation was carried out in the dark at 23 °C for 40 min with gentle inversion (approximately 4 rpm) as previously described [43]. Microarray slides were incubated with PKH26-labelled EVs diluted in buffer containing 20 mM Tris-HCl, 100 mM NaCl, 1 mM CaCl_2_, 1 mM MgCl_2_ (pH 7.4) with 0.025% Tween 20 (TBST) for 40 min at 23 °C. Titrations with labeled EVs were carried out at 5, 2.5, 1.25, and 0.63 μg/mL. Subsequent lectin microarray interrogations were performed in 12 replicates, four from one EV isolation sample (*Sm*EV1 A–D) and eight from pooled EV samples (*Sm*EVc A–H), at 3 µg/mL which provided the best signal to noise ratio and fewest artefacts. Following incubation, arrays were unpacked under TBST, washed once in TBST for 5 min with gentle shaking and then briefly rinsed with TBS before being centrifuged dry (450× *g*, 5 min). Microarrays were imaged immediately at 532 nm in an Agilent G2505B microarray scanner at 100% PMT. Data extraction was carried out in GenePix Pro (v6.0, Molecular Devices, San Jose, CA, USA), processing was carried out in Excel (v2013, Microsoft, Redmond, WA, USA), and analysis performed with Excel, HCE 3.5 (http://www.cs.umd.edu/hcil/hce/, accessed on 13 April 2018) and Morpheus (Morpheus, https://software.broadinstitute.org/morpheus, accessed on 28 May 2021).

### 4.5. Neuraminidase Treatment

Neuraminidase treatments employed a broad-spectrum α2-3,6,8,9 neuraminidase A (New England Biolabs P0722, Ipswich, MA, USA). For microarray experiments, no detergents or reducing agents were used in the enzymatic digestion and hydrolysis was carried out on PKH26-labeled EVs in the dark at 37 °C for 1.5 h. For Western blots and free sialic acid detection assays, 50 μg of proteins (EV samples or SWAP) were incubated with 40 units (2 μL) neuraminidase for 1.5 h at 37 °C according to the manufacturer’s protocol and assayed immediately. 10 μg fetuin (New England Biolabs P0722, Ipswich, MA, USA) was used as a control sialylated glycoprotein.

### 4.6. Free Sialic Acid Detection Assay

Free sialic acid was quantified according to the manufacturer’s instruction using a Sialic Acid Assay Kit (abcam ab83375, Cambridge, UK), which uses an enzyme coupled reaction in which oxidation of free sialic acid creates an intermediate that reacts stoichiometrically with a probe, generating a product which is detected by measuring fluorescence (Ex/Em = 535/587 nm) using a Synergy H4 Hybrid plate reader.

### 4.7. Lectin- and Immunofluorescence Microscopy of Parasites

Dextran-labelled parasites were prepared as previously described [29]. Briefly, ~50 freshly perfused worms were immersed in 100 μL of a 5 mg/mL solution of biotin-TAMRA-dextran (Life Technologies D3312) dissolved in ultrapure water. Worms were gently vortexed for 3 min, and then submerged in 10 mL fixative solution (4% formaldehyde in PBSTx (PBS + 0.3% triton-X100)) to stop labeling. The fixative solution was discarded and replaced with 10 mL fresh fixative solution to dilute residual dextran. The worms were subsequently fixed for 4 h in the dark with mild agitation. Parasites were subsequently washed with 10 mL fresh PBSTx for 10 min and then labelled with FITC-conjugated lectins as detailed below. In other experiments, adult *S. mansoni* worms were flat-fixed in 4% paraformaldehyde (PFA) in PBS; freshly perfused male and female worms were separated, placed between two microscope slides and further flattened under additional weight while submerged in 4% PFA in the dark for 4 h at 4 °C. Following fixation, worms were washed in antibody diluent (AbD; 0.1% BSA, 0.1% NaN_3_, 0.5% Triton x-100 in 1X PBS) and incubated with biotinylated lectins (SNA-I, DSA, or RCA-I; 1:100 in AbD) (Biolynx B-1305, B-1185, B-1085, Brockville, ON, Canada) for 3 days at 4 °C. Parasites were then washed in AbD overnight and incubated with fluorescein (FITC)-conjugated anti-biotin (abcam ab53469, Cambridge, UK) diluted 1:500 in AbD for 3 days at 4 °C. Worms were again washed in AbD overnight and counterstained overnight with tetramethylrhodamine (TRITC)-conjugated phalloidin (Millipore Sigma, Oakville, ON, Canada) (1:100 in AbD) at 4 °C, after which parasites were washed in AbD overnight and mounted on glass microscope slides with Vectashield^®^ anti-fading solution (Vector Laboratories). Specimens were viewed using a Leica TCS SP5 confocal scanning microscope or a Nikon A1R MP confocal scanning microscope.

### 4.8. Fluorescence In Situ Hybridisation (FISH) Lectin Co-Labelling

Fluorescence in situ hybridisation was performed as described [29,44,45]. To label the cytoplasm of tegumental cells by FISH, riboprobes recognising the tegument-specific markers *calpain*, *gtp-4*, *annexin*, and *npp-5* were pooled as described by Wendt et al., 2018. Male and female parasites were separated by incubation (2–3 min) in 0.25% ethyl 3-aminobenzoate methanesulphonate (Sigma-Aldrich A5040) in PBS. Relaxed worms were fixed for 4 h in the dark in 4% formaldehyde in PBSTx (PBS + 0.3% triton-X100) with gentle agitation. Following the fixation process, parasites were dehydrated in methanol and rehydrated in 1:1 methanol:PBSTx followed by incubation in PBSTx. Rehydrated worms were then bleached under bright light for 1 h in formamide bleaching solution (0.5% formamide, 0.5% saline-sodium citrate buffer, and 1.2% H_2_O_2_), rinsed with PBSTx and briefly post-fixed for 10–15 min in 4% formaldehyde in PBSTx. Samples were hybridised overnight with the 4 riboprobes at 52 °C. Worms were then washed in AbD and incubated with fluorescein-conjugated lectins (SNA-I or DSA) (Vector Laboratories, FL-1301-2 and FL-1181-2, Burlingame, CA, USA); 4 μg in 500 μL AbD) overnight at 4 °C. FISH-labeled parasites were exposed to either FITC-conjugated SNA-I or DSA as described above, counterstained with DAPI (1 mg/mL), cleared in 80% glycerol, and mounted on slides with Vectashield (Vector Laboratories). Specimens were viewed using a Nikon A1R MP confocal scanning microscope.

## Figures and Tables

**Table 1:** Lectins exhibiting strong adhesion with *Schistosoma mansoni* EVs with specificity information for each.

Lectin	Organism	Common Name	Specificity
SNA-II	*Sambucus nigra*	Sambucus lectin-II	Gal/GalNAc
DSA	*Datura stramonium*	Jimson weed lectin	GlcNAc
LEL	*Lycopersicum eculentum*	Tomato lectin	GlcNAc-β-(1,4)-GlcNAc
Calsepa	*Calystegia sepium*	Bindweed lectin	Man/Maltose
NPA	*Narcissus pseudonarcissus*	Daffodil lectin	α-(1,6)-Man
GNA	*Galanthus nivalis*	Snowdrop lectin	Man-α(1,3)-
HHA	*Hippeastrum hybrid*	Amarylis agglutinin	Man-α(1,3)-Man-α(1,6)-
WGA	*Triticum vulgaris*	Wheat germ agglutinin	NeuAc/GlcNAc
SNA-I	*Sambucus nigra*	Sambucus lectin-II	Sialic acid-α-(2,6)-Gal(NAc)
PHA-E	*Phaseolus vulgaris*	Kidney bean erythroagglutinin	biantennary, bisecting GlcNAc,β-Gal/Gal-β-(1,4-)GlcNAc
RCA-I/120	*Ricinus communis*	Castor bean lectin	Gal-β-(1,4)-GlcNAc
CAA	*Caragana arborescens*	Pea tree lectin	Gal-β-(1,4)-GlcNAc

**Table 2:** Summary of the effect of neuraminidase treatment on lectin affinity.

Difference ^1^	*p* Value	Significance ^2^	+/−? ^1^	Lectin	Top Ligands
11,386.58	0.000285	***	Up	SNA-II	Gal/GalNAc
2829.56	0.028967	*	Down	LEL	GlcNAc-β-(1,4)-GlcNAc
775.70	0.000462	***	Down	Calsepa	Man/Maltose
5911.46	0.001086	***	Down	WGA	NeuAc/GlcNAc
8346.68	0.000001	****	Down	SNA-I	Sialic acid-α-(2,6)-Gal(NAc)
1610.86	0.003570	**	Down	PHA-E	biantennary, bisecting GlcNAc,β-Gal/Gal-β-(1,4-)GlcNAc
8081.83	0.009170	**	Up	RCA-I	Gal-β-(1,4)-GlcNAc
858.01	0.002417	**	Up	ECA	Gal-β-(1,4)-GlcNAc oligomers

^1^ Numbers in red show an increase in lectin affinity (Up) following incubation with neuraminidase, whereas numbers in green represent a decrease in lectin affinity (Down) following enzymatic digestion. ^2^ Statistics were performed using a paired *t*-test * *p* > 0.05, ** *p* > 0.01, *** *p* > 0.0005, **** *p* > 0.00001.

## Data Availability

All the data presented in this study are available in this manuscript.

## Supplementary Materials

The following are available online at https://www.mdpi.com/article/10.3390/pathogens10111401/s1, Table S1: Lectin array v2.4.0 print list with coarse specificity information for each lectin.
