# Supplementary material for: Analysis of Schistosoma mansoni Extracellular Vesicles Surface Glycans Reveals Potential Immune Evasion Mechanism and New Insights on Their Origins of Biogenesis
Source: Pathogens. 2021 Oct 29;10(11):1401. doi: 10.3390/pathogens10111401 (PMC8617790; doi:10.3390/pathogens10111401)
Supplement: Supplementary file 1 [file pathogens-10-01401-s001.zip › pathogens-1422273-supplementary.pdf]

No	Lectin	Organism	Common name	Specificity
1	AIA Jacalin	*Artocarpus intergrifolia*	Jack fruit lectin	Gal (sialylation tolerant)
2	RPbAI	*Robinia pseudoacacia*	Black locust lectin	Gal, GalNAc
3	SNA-II	*Sambucus nigra*	Sambucus lectin-II	Gal/GalNAc
4	SJA	*Sophora japonica*	Pagoda tree lectin	β-GalNAc
5	DBA	*Dolichos biflorus*	Horse gram lectin	GalNAc
6	GHA	*Glechoma hederacea*	Ground ivy lectin	GalNAc
7	SBA	*Glycine max*	Soy bean lectin	GalNAc
8	VVA	*Vicia villosa*	Hairy vetch lectin	GalNAc
9	BPA	*Bauhinia purpurea*	Camel's foot tree lectin	GalNAc/Gal
10	WFA	*Wisteria floribunda*	Japanese wisteria lectin	GalNAc/sulfated GalNAc
11	HPA	*Helix pomatia*	Garden snail lectin	α-GalNAc
12	GSL-I-A4	*Griffonia simplicifolia*	Griffonia lectin-I A4	GalNAc
13	ACA	*Amaranthus caudatus*	Amaranthin	Sialylated/Gal-β-(1,3)-GalNAc
14	ABL	*Agaricus bisporus*	Edible mushroom lectin	Gal-β-(1,3)-GalNAc, GlcNAc
15	PNA	*Arachis hypogaea*	Peanut lectin	Gal-β-(1,3)-GalNAc
16	GSL-II	*Griffonia simplicifolia*	Griffonia /Bandeiraea lectin-II	GlcNAc
17	sWGA	*Triticum vulgaris*	Succinyl WGA	GlcNAc
18	DSA	*Datura stramonium*	Jimson weed lectin	GlcNAc
19	STA	*Solanum tuberosum*	Potato lectin	GlcNAc oligomers
20	LEL	*Lycopersicum eculentum*	Tomato lectin	GlcNAc-β-(1,4)-GlcNAc
21	Calsepa	*Calystegia sepium*	Bindweed lectin	Man/Maltose
22	NPA	*Narcissus pseudonarcissus*	Daffodil lectin	α-(1,6)-Man
23	GNA	*Galanthus nivalis*	Snowdrop lectin	Man-α(1,3)-
24	HHA	*Hippeastrum hybrid*	Amarylis agglutinin	Man-α(1,3)-Man-α(1,6)-
25	ConA	*Canavalia ensiformis*	Jack bean lectin	Man, Glc, GlcNAc
26	Lch-B	*Lens Culinaris*	Lentil isolectin B	Man, fucose dependent
27	Lch-A	*Lens Culinaris*	Lentil isolectin A	Man, fucose dependent
28	PSA	*Pisum sativum*	Pea lectin	Man, fucose dependent
29	TJA-I	*Trichosanthes japonica*	TJA One	Sialic acid-α-(2,6)-Gal(NAc)
30	WGA	*Triticum vulgaris*	Wheat germ agglutinin	NeuAc/GlcNAc
31	MAA	*Maackia amurensis*	Maackia agglutinin	Sialic acid-α-(2,3)-Gal(NAc)
32	SNA-I	*Sambucus nigra*	Sambucus lectin-II	Sialic acid-α-(2,6)-Gal(NAc)
33	CCA	*Cancer antennarius*	California crab	9-O -acetyl-sialic acid
34	PHA-L	*Phaseolus vulgaris*	Kidney bean leukoagglutinin	tri-/tetra-antennary β-Gal/Gal-β-(1,4)-GlcNAc
35	PHA-E	*Phaseolus vulgaris*	Kidney bean erythroagglutinin	biantennary, bisecting GlcNAc,β-Gal/Gal-β-(1,4-)GlcNAc
36	RCA-I/120	*Ricinus communis*	Castor bean lectin	Gal-β-(1,4)-GlcNAc
37	AMA	*Arum maculatum*	Lords and Ladies agglutinin	Gal-β-(1,4)-GlcNAc
38	CPA	*Cicer arietinum*	Chickpea lectin	Complex glycopeptides
39	CAA	*Caragana arborescens*	Pea tree lectin	Gal-β-(1,4)-GlcNAc
40	ECA	*Erythrina cristagalli*	Cocks comb/coral tree lectin	Gal-β-(1,4)-GlcNAc oligomers
41	TJA-II	*Trichosanthes japonica*	TJA Two	Fuc-α(1,2)Gal(NAc)-β(1,4)
42	AAL	*Aleuria aurantia*	Orange peel fungus lectin	α-(1,6 and 1,3)-linked Fuc
43	LTA	*Lotus tetragonolobus*	Lotus lectin	α-(1,3)-linked Fuc
44	UEA-I	*Ulex europaeus*	Gorse lectin-I	α-(1,2)-linked Fuc
45	PA-I	*Pseudomonas aeruginosa*	Pseudomonas lectin	α-Gal, Gal derivatives
46	EEA	*Euonymous europaeus*	Spindle tree lectin	α-Gal
47	GSL-I-B4	*Griffonia simplicifolia*	Griffonia /Bandeiraea lectin-I	α-Gal
48	MPA	*Maclura pomifera*	Osage orange lectin	α-Gal
49	VRA	*Vigna radiata*	Mung bean lectin	α-Gal
50	MOA	*Marasmius oreades*	Fairy ring mushroom lectin	α-Gal
51	**PBS**	N/A	N/A	N/A
52	**BSA**	N/A	N/A	N/A

Supplementary Table S1: Lectin array v2.4.0 print list with coarse specificity information for each lectin.
